# The Development of Mandarin-Speaking Children’s Comprehension of Irony

**DOI:** 10.3390/bs16101803

**Published:** 2026-10-02

**Authors:** Ming Zhao, Ying Jiang, Xiaosong Gai

**Affiliations:** 1 School of Education, Jilin International Studies University, Changchun 130117, China; jisuzhaoming@126.com; 2 College of Education, Dalian University, Dalian 116622, China; jiangying@dlu.edu.cn; 3 School of Psychology, Northeast Normal University, Changchun 130024, China; 4 Key Research Base of Humanities and Social Sciences of Higher Education in Jilin Province, Center of Mental Health Education and Research, Northeast Normal University, Changchun 130024, China

**Keywords:** irony, speaker attitude, meaning inference, error types, pragmatic development

## Abstract

This study examined age-related differences in irony comprehension among 168 Mandarin-speaking children aged 6–10 years. Each child watched four illustrated stories with prerecorded audio: one literal, one deception, and two irony stories. For each irony story, children judged the speaker’s satisfaction, identified praise or criticism, and explained the remark. Their answers were then classified into four ordered performance levels. Age-group and irony-condition effects were tested with a generalized ordinal GEE clustered by child, and explanation errors were examined descriptively. Performance differed significantly by age group but not by irony condition. Six-year-olds identified the speaker’s dissatisfaction and intended criticism in 40.4% of their classifiable story responses, compared with 83.3% among 10-year-olds. Yet only about two in five responses from 10-year-olds included a correct or near-correct explanation. Children who misjudged both attitude and meaning usually understood the remark literally and treated it as sincere. When children recognized dissatisfaction and criticism but could not explain the irony, most responses contained no relevant explanation, while some explained the remark as an attempt to conceal dissatisfaction or avoid direct criticism. Together, these findings show age-related differences in recognizing ironic criticism and in performance on the explanation task. They also suggest that some awareness of the speaker’s strategy may be present before children can explain irony adequately.

## 1. Introduction

Verbal irony involves using an utterance to convey a meaning or evaluative attitude that contrasts with what its literal wording appears to express in context (Dews et al., 1996; Köder & Falkum, 2021). For example, after someone arrives extremely late to a meeting, “You are really punctual” may convey criticism rather than praise. The contrast need not be a word-for-word reversal of sentence meaning; an apparently appreciative remark may also convey dissatisfaction in context.

Understanding irony requires children to relate an utterance to its context and infer the speaker’s attitude and communicative intention. A literal interpretation can obscure criticism, humor, or other information relevant to a social interaction. Irony also occurs in instructional materials and children’s media, making its development relevant to the choice of language used with children (Lazar et al., 1989; Weaver et al., 1988).

Early educational studies illustrated the possible consequences of difficulty with irony. Cantor and Reilly (1979) found that humor closely related to instructional content, particularly irony, was associated with poorer learning among children. Research on educational television also found that irony could lead children to misunderstand information and that subsequent corrections did not always eliminate the misunderstanding (Zillmann et al., 1984; Weaver et al., 1988). For example, ironic descriptions of unfamiliar objects could leave children with inaccurate impressions of their properties. These findings show that instructional irony can interfere with learning under some conditions. They do not establish that reduced learning was caused solely by a failure to recognize irony; attention, memory, and the demands of learning unfamiliar content may also have contributed.

Developmental studies have used different criteria for successful irony comprehension. In an English-language study, Ackerman (1983) presented first- and third-grade children, approximately six and eight years old, with short stories and asked questions about the situation and the speaker’s attitude. In the first experiment, each child age group comprised 36 participants. Under a favorable combination of contextual and intonational cues, approximately 53% of the younger children’s and 72% of the older children’s attitude judgments were correct. These figures indicate sensitivity to the speaker’s attitude under those conditions; they should not be read as overall comprehension rates across all conditions.

Dews et al. (1996) used different materials and questions. In Study 1, 89 English-speaking participants comprising 5- to 6-year-olds, 8- to 9-year-olds, and adults watched cartoon scenarios featuring ironic criticisms, literal criticisms, and literal compliments. Participants selected speakers’ intended meanings and judged how mean and how funny the remarks were. Study 2 involved 135 participants, including 6- to 7-year-olds, 8- to 9-year-olds, and adults, and examined the effects of utterance form and intonation. Judgments of meaning, meanness, and humor did not develop in parallel. More generally, children younger than six often show limited and variable performance on conventional irony-comprehension tasks. This is a statement about performance under particular testing conditions, rather than a categorical limit on younger children’s competence.

For older children, estimates of irony comprehension also depend on what a task requires. Ackerman’s questions assessed children’s interpretation of speaker attitude. Demorest et al. (1983) instead asked children to explain both the discrepancy between figurative remarks and the facts and the speaker’s communicative purpose. Their eight-year-olds often noticed the discrepancy without explaining its purpose, whereas eleven-year-olds were more likely to identify both. Taken together, these studies make clear why attitude detection should not be equated with a full account of an ironic utterance. Differences between tasks, however, do not by themselves establish successive developmental stages.

The present study examines how attitude detection, meaning inference, and explanation occur together in children’s responses and how performance on these measures varies with age. Recognizing a speaker’s dissatisfaction may help a child infer that an apparently positive remark is intended as criticism, but it does not guarantee an accurate inference or explanation. Response patterns across the three measures may be consistent with differing degrees of understanding. The resulting levels are treated as ordered performance categories on the present tasks, not fixed stages through which every child must pass. Because offline judgment and explanation tasks place demands on children’s language and response abilities, these categories must be interpreted with these demands in mind (Fuchs, 2023).

A second question is how children interpret irony when their understanding is incomplete. Do they treat the utterance as sincere, as a mistake, or as an attempt to deceive? Demorest et al. (1984) asked children to judge whether the utterance was true, whether the speaker believed it, and whether the speaker wanted the listener to believe it. Approximately 72% of the children’s responses to sarcastic remarks were classified as deceptive interpretations. Capelli et al. (1990) asked children to explain speakers’ intentions in sarcastic stories and found that, among responses in which children failed to detect the sarcasm, 68% of third-graders’ and 72% of sixth-graders’ responses were classified as sincere interpretations, whereas 22% in each age group were classified as lying. Because Demorest et al. (1984) and Capelli et al. (1990) used different questions, coding procedures, and response denominators, these percentages should not be treated as directly comparable estimates of the same error rate. Their contrasting patterns nevertheless raise a question about what children take the speaker to be doing when they fail to understand irony, and whether the errors differ with the degree of understanding children demonstrate.

Hayashi and Ban (2021) found that younger children sometimes judged speakers in ironic situations to be intending praise and listeners to be hearing praise. Performance was associated with second-order false-belief understanding, particularly in situations involving unintended or unsuccessful irony. This association does not establish a single cause of children’s errors; pragmatic experience, language ability, and task demands may also be relevant. A sincere interpretation is especially informative when it conflicts with the facts given in a story. Children’s explanations may show whether they rely on the literal wording, reinterpret the facts, or assign another purpose to the speaker. Examining such responses can identify possible difficulties in irony comprehension, although it cannot by itself establish their causes.

Finally, pragmatic understanding develops within particular linguistic and social contexts. Findings from children studied in other languages cannot simply be assumed to apply in full to Mandarin-speaking children. Research on Chinese-speaking children’s interpretation of indirect utterances remains limited (Miao, 1995), and broad parent-report measures of Mandarin pragmatic development do not directly assess irony comprehension (Qian et al., 2022). A comparison of Chinese and American adults found that Chinese participants rated sarcastic comments as less sarcastic than American participants did (Du et al., 2024), but adult findings cannot establish a distinct developmental pattern in children. The present study extends research on irony comprehension to Mandarin-speaking children without presupposing such a pattern. It addresses two questions:

RQ1. What age-related patterns characterize irony comprehension among Mandarin-speaking children?

RQ2. What types of errors do Mandarin-speaking children make when interpreting irony, and what do these errors reveal about the difficulties underlying irony comprehension?

## 2. Method

### 2.1. Participants

Sample size was determined a priori using G*Power 3.1. Assuming an effect size of f = 0.30, with α = 0.05 and power = 0.80, an F-test approximation for the between-group age effect across four age groups indicated that a minimum total sample size of 126 was required. A total of 168 Mandarin-speaking children participated in the study. They were recruited from two ordinary primary schools and two kindergartens in Changchun, China. The participating institutions were identified through convenience sampling. Study information was circulated via WeChat (version 8.0.49), and administrators of interested schools and kindergartens contacted the research team to arrange data collection. Children were eligible for inclusion if, according to parental or guardian report, they had no known hearing, speech, language, intellectual, or developmental disorders.

The sample comprised four approximate age groups, referred to throughout as the 6-, 7-, 8-, and 10-year-old groups; exact mean ages and age ranges are reported in Table 1. The 6- to 8-year-old groups were sampled at one-year intervals because previous studies have identified this period as one of substantial change in performance on irony-comprehension tasks (Ackerman, 1983; Demorest et al., 1983; Dews et al., 1996). A 10-year-old group, half the size of each younger age group, was included as an older comparison group. Because the study was designed to compare selected developmental points rather than to trace a complete year-by-year trajectory, no 9-year-old group was included. Recruitment was balanced by sex within each age group. The distribution of participants is shown in Table 1.

### 2.2. Materials

Studies of children’s irony comprehension often include literal and deception stories alongside irony stories (Ackerman, 1981; Sullivan et al., 1995; Winner & Leekam, 1991). In a literal story, the target utterance is consistent with the facts. For example, when the homework took six hours, the mother said “That took a lot of effort, didn’t it?” (Ackerman, 1983). In a deception story, the speaker says something inconsistent with the facts but intends the listener to believe it. For example, Simon had not cleaned his room and did not know that his grandmother had seen the mess, yet he said “I did a really good job cleaning up my room” (Sullivan et al., 1995). In the irony stories used here, an apparently positive utterance contrasts with a negative outcome and is not intended as sincere praise or thanks. The literal and deception stories helped distinguish interpretation of the ironic utterances from a general tendency to choose a meaning opposite to the literal wording.

Accordingly, the experimental materials in the present study consisted of four stories: a literal story, a deception story, an assertive-type irony story, and an attitudinal-type irony story. In the literal story, the literal sentence meaning of the utterance matched the intended speaker meaning. For example:

“Xiaohong was playing with building blocks. She had built a very tall house out of blocks. Xiaoming came over and said to Xiaohong, ‘I will add one more block.’ After Xiaoming spoke, he added another block on top. Now Xiaohong’s block house was even more beautiful than before. Xiaohong said to Xiaoming, ‘You did a great job.’”

In the assertive-type irony story, the outcome of Xiaoming’s action was changed to “As a result, Xiaohong’s block house collapsed,” while Xiaohong’s final utterance remained “You did a great job.” In the attitudinal-type irony story, the same negative outcome was followed by “Thank you very much.” The labels are informed by the speech-act distinction between assertions about states of affairs and expressions of a speaker’s psychological state, such as gratitude (Searle, 1976), and describe the apparent forms of these two utterances. Holding the negative outcome constant while varying the apparent speech-act form of the final utterance allowed us to compare children’s responses to an apparent positive evaluation (“You did a great job”) and apparent gratitude (“Thank you very much”). The deception story had a related plot and characters, but involved an attempt to mislead the listener.

All stories were kept under 150 Chinese characters. Full versions of the stories are provided in Text S1 of the Supplementary Materials.

The story audio was prerecorded by a child narrator, who was instructed to read the protagonists’ dialogue naturally, as if reading a story. The recorded audio and four accompanying pictures for each story were integrated in Microsoft PowerPoint 2021 and presented on a laptop computer. For each story, all children heard the same recording and saw the same pictures. In the final picture, the speaker was shown from a non-frontal angle (i.e., an oblique view rather than a full frontal view of the face) to reduce the availability of facial-expression cues.

For each of the two irony stories, children answered the following questions in the order shown:

1. Self-reported comprehension: Did you understand the story?
2. Fact memory question: What did Xiaoming (the addressee) do?
3. Utterance memory question: What was the last sentence in the story?
4. Attitude detection: Was Xiaohong (the speaker) satisfied with Xiaoming (the addressee)?
5. Meaning inference: Was Xiaohong praising Xiaoming or criticizing him?
6. Explanation: Why did she (the speaker) say that?

Question 1 served as a preliminary self-report check of comprehension, whereas Questions 2 and 3 assessed recall of the relevant action and target utterance. A story was replayed if a child had difficulty recalling its facts or target utterance before proceeding to the interpretation questions. Children also answered comprehension and interpretation questions about the literal and deception stories; because the speaker and listener could differ between stories, the wording above illustrates the questions for the irony stories rather than a verbatim script for all four stories.

The construction of Questions 4–6 was informed by previous developmental research distinguishing speaker attitude, intended utterance meaning, and explicit explanations of nonliteral utterances (Ackerman, 1983; Andrews et al., 1986; Demorest et al., 1983). Question 4 assessed whether children identified the speaker’s dissatisfaction, Question 5 whether they inferred praise or criticism, and Question 6 elicited an open-ended explanation. In both irony stories, “dissatisfied” and “criticizing” were the target responses to Questions 4 and 5, respectively. In the “Thank you very much” story, “criticizing” referred to an inferred negative evaluation of the addressee’s action.

Prior to testing the children, the story materials underwent a content-validity check with 48 third-year university students in a group paper-and-pencil pilot. All participants’ responses were consistent with the intended story types. For the literal and deception stories, they interpreted the target utterances literally and provided corresponding explanations. For the two irony stories, they gave ironic interpretations and explained the utterances using terms such as “irony,” “saying the opposite,” or “speaking ironically.” Accuracy was 100% for attitude detection, meaning inference, and explanation across all four stories. These results indicate that the materials elicited the intended interpretations in the adult sample.

### 2.3. Procedure

Children were tested individually in a quiet classroom. Each child completed all four stories in a single session lasting approximately 15 min. The instructions were as follows:

“Today the teacher will show you some stories. After each story, you will be asked several questions. You have to listen carefully so that you can answer the questions correctly. Are you ready? Let’s begin.”

The literal and deception stories were always presented before the two irony stories to familiarize children with the experimental procedure. Within each age group, the order of the two irony stories was counterbalanced: half of the children received the assertive-type irony story first, and the other half received the attitudinal-type irony story first.

### 2.4. Scoring and Coding

Responses to the attitude-detection and meaning-inference questions were each scored as correct or incorrect. Explanations were coded as correct, near-correct, incorrect, or no explanation. Correct explanations explicitly identified irony or the discrepancy between the utterance’s apparent meaning and the speaker’s intended meaning. Near-correct explanations referred to the speaker’s negative emotion or marked tone without explicitly identifying that discrepancy. Responses that provided a reason but met neither criterion were coded as incorrect, whereas failure to provide a reason was coded as no explanation.

Two authors independently coded 84 of the 336 explanation responses, randomly selected across age groups (approximately one-quarter). Inter-coder agreement for the four explanation categories (correct, near-correct, incorrect, and no explanation) ranged from 88% to 100%; for the near-correct category specifically, both authors evaluated 5 responses and agreed on all 5 (100%). Disagreements were resolved through discussion until full agreement was reached. The remaining 252 responses were coded by one author according to the established criteria.

### 2.5. Statistical Analysis

Performance on the literal and deception stories was summarized descriptively. To examine response consistency across the two irony conditions, responses to the attitude-detection and meaning-inference tasks were each cross-tabulated in a 2 × 2 table, with each child contributing one paired observation to each table. Pearson’s chi-square test of independence, without Yates’s continuity correction, was used to test the null hypothesis that the response category in one irony condition was independent of the corresponding response category in the other condition. The diagonal cells represented consistent response categories, whereas the off-diagonal cells represented inconsistent response categories.

To address RQ1, performance on attitude detection, meaning inference, and explanation, together with the distribution of the four response levels, was summarized by age group. Age-group differences in response levels were then examined using a generalized ordinal GEE; irony condition and the age-group-by-condition interaction were also evaluated. To address RQ2, explanations accompanying Level 0–2 responses were classified into five error types, and their frequencies and within-level percentages were reported.

The generalized ordinal GEE analyses were conducted in Python 3.12.14 using statsmodels 0.15.0 (Seabold & Perktold, 2010). Each irony response was coded on a four-level ordinal scale (Levels 0–3). Because each child contributed one response in each irony condition, child was specified as the clustering unit. The ordinal outcome was represented by three cumulative indicators—at least Level 1, at least Level 2, and Level 3—and analyzed using binomial generalized estimating equations with a logit link and an independence working correlation structure.

Nested model comparisons were conducted using robust GEE score tests evaluated under the corresponding constrained models. These tests were used to assess the proportional-odds assumption and the age-group-by-condition interaction. Because the proportional-odds assumption was not supported, the final generalized ordinal GEE allowed the effects of age group and irony-story condition (assertive-type vs. attitudinal-type) to vary across the three cumulative thresholds. The joint age-group-by-condition interaction did not reach the 0.05 significance level; therefore, the final model included threshold-specific age-group and irony-condition effects without their interaction.

For inference from the final fitted model, participant-clustered bias-reduced sandwich covariance estimation was used (Mancl & DeRouen, 2001). Overall age-group and irony-condition effects were evaluated using joint Wald tests across thresholds. Pairwise age-group differences were also tested jointly across thresholds, with Bonferroni correction applied to the six comparisons. Threshold-specific odds ratios and 95% confidence intervals were reported as effect-size estimates. Two responses that could not be assigned a comprehension level were omitted from this analysis; the other response provided by each of these two children was retained.

To assess sensitivity to the treatment of near-correct explanations, the final generalized ordinal GEE model was refitted after the 19 responses containing such explanations were reassigned from Level 3 to Level 2.

## 3. Results and Analyses

### 3.1. Preprocessing of the Experimental Data

#### 3.1.1. Consistency of Responses Across the Two Irony Stories

Children’s response stability was assessed on the basis of the consistency of their responses across the two irony conditions. A response was considered consistent when a child’s answers under the two irony conditions fell into the same response category—that is, “satisfied” or “dissatisfied” for the attitude-detection task and “praising” or “criticizing” for the meaning-inference task—including cases in which both responses were correct and cases in which both were incorrect. All other response combinations were coded as inconsistent. For the attitude-detection task, the full observed 2 × 2 table, with the assertive-type response listed first and the attitudinal-type response second, contained 50 satisfied–satisfied, 8 satisfied–dissatisfied, 8 dissatisfied–satisfied, and 102 dissatisfied–dissatisfied paired responses. Thus, 152 of the 168 paired responses were consistent and 16 were inconsistent; Pearson’s chi-square test indicated a significant association between responses across the two irony conditions, χ^2^ (1, *n* = 168) = 104.67, *p* < 0.001. For the meaning-inference task, the corresponding counts were 80 criticizing–criticizing, 8 criticizing–praising, 11 praising–criticizing, and 69 praising–praising. Thus, 149 paired responses were consistent and 19 were inconsistent; Pearson’s chi-square test likewise indicated a significant association, χ^2^ (1, *n* = 168) = 100.49, *p* < 0.001. These results indicate statistically significant associations between responses across the two irony conditions, but they do not by themselves establish genuine understanding or rule out a stable response bias.

#### 3.1.2. Comprehension and Memory Checks

We examined the 168 children’s self-reported comprehension of the stories and their memory for the relevant facts and target utterances. One child reported not understanding the attitudinal-irony story despite correctly recalling the relevant facts and target utterance. In addition, six participants initially made errors when recalling the speaker’s utterance in the literal story, and one participant made an error when recalling the speaker’s utterance in an irony story. After one repetition, all seven children correctly recalled the target utterance.

Apart from these cases, the other participants reported understanding the stories and recalled the relevant facts and target utterances. These checks suggest that most children retained the information needed for the interpretation questions.

#### 3.1.3. Preliminary Description of the Results

Analyses of the literal and deception stories showed that performance was at ceiling for the literal story in all age groups. In the deception story, all children in the three older groups responded correctly, while 43 of the 48 children in the youngest group (89.6%) correctly identified the intended meaning. Thus, deception was generally well understood, although a small proportion of the youngest children still experienced difficulty.

Under the irony story conditions, Table 2 presents the percentages of individual story responses meeting the criteria for attitude detection, meaning inference, and explanation in each age group. For the explanation task, examples of responses corresponding to these coding categories included correct reasons such as “She is saying the opposite,” “She is using an opposite word,” “It is sarcasm,” and “‘You did a great job’ actually means something bad.” Near-correct reasons included responses such as “She is very angry” and “She said it in an angry way.” Such responses were treated as partial evidence that the child had detected the speaker’s negative affect or marked delivery. Previous research has shown that children’s explanations of ironic remarks often refer to speakers’ emotions or intentions (Garfinkel et al., 2024) and that emotion understanding is associated with children’s interpretation of ironic intent (Nicholson et al., 2013). Correct and near-correct explanations were therefore grouped as meeting the explanation criterion in the primary analyses and when calculating the percentages in Table 2; the effect of this decision was examined in a sensitivity analysis reported in Section 3.3. The remaining responses contained either an incorrect explanation or no explanation.

These results show that, among individual irony-story responses, attitude detection was more frequent than meaning inference at ages 6 and 7 (59% vs. 42% and 52% vs. 44%, respectively). Among 8-year-olds, 75% of responses met the attitude-detection criterion and 59% met the meaning-inference criterion. Among 10-year-olds, the corresponding proportions were 85% and 83%. The explanation criterion was met less often in all age groups (2%, 19%, 16%, and 40%, respectively); even among 10-year-olds, fewer than half of responses included a correct or near-correct explanation. The numbers of correct and near-correct explanations, respectively, were 1 and 1 among 6-year-olds, 16 and 2 among 7-year-olds, 6 and 9 among 8-year-olds, and 12 and 7 among 10-year-olds.

### 3.2. Classification of Responses into Four Performance Levels

Based on the relationship between speaker attitude and utterance meaning in irony comprehension, we hypothesized that judgments about the meaning of irony (whether the utterance is a criticism or a compliment) are typically based on recognition of the speaker’s attitude (whether the speaker is satisfied or dissatisfied). Thus, in tasks involving irony, attitude detection may constitute a more basic component of meaning inference. The descriptive statistics above also showed that, in every age group, the attitude-detection criterion was met more often than the meaning-inference criterion. We therefore further hypothesized that attitude detection may reflect a lower level of irony comprehension, whereas meaning inference may reflect a higher level.

If this hypothesis is correct, then (1) participants who succeed on meaning inference should also succeed on attitude detection, and (2) participants who fail on attitude detection should not succeed on meaning inference. To test these predictions, we compared participants’ responses on the irony attitude detection and meaning inference tasks. Among the 179 correct responses on the meaning inference task, 98.9% were also correct on the attitude detection task. Among the 116 failures on the attitude detection task, only 1.7% were correct on the meaning inference task.

Of the 45 response combinations that were inconsistent across the two tasks, 43 consisted of correct responses on the attitude detection task but incorrect responses on the meaning inference task; only two response combinations, both from children in the 6-year-old group, were incorrect on attitude detection but correct on meaning inference. This pattern supports our expectation and indicates that correct meaning inference in irony is typically accompanied by correct attitude detection. Attitude detection thus represents a relatively lower-level performance in irony comprehension, whereas meaning inference represents a relatively higher-level performance.

Even among participants who correctly inferred the meanings of the ironic utterances, performance on the subsequent explanation task varied. Of the 179 correct meaning inference responses, only 30.2% were accompanied by correct or near-correct reasons; 31.9% were accompanied by incorrect explanations, and 38% were accompanied by no explanation. It should be noted that, compared with Level 2, Level 3 may also partly reflect children’s ability to verbalize their pragmatic knowledge.

On the basis of these analyses, we distinguished four levels of children’s comprehension of irony, as shown in Table 3.

### 3.3. Performance Levels by Age Group and Irony Condition

Using the classification criteria presented in Table 3, each irony response was assessed for classification as Level 0, 1, 2, or 3. Of the 336 potential responses contributed by 168 children, 334 could be classified. Two attitudinal-irony responses from two 6-year-old children could not be assigned a level: in both cases, the child judged the speaker to be satisfied while correctly identifying the utterance as a criticism. These were the two responses excluded from the GEE analysis. Descriptive statistics for the classified irony responses are presented in Table 4.

The proportional-odds assumption was not supported, robust score χ^2^ (8) = 16.02, *p* = 0.042. The final GEE model therefore allowed age-group and irony-condition effects to vary across the three cumulative thresholds. The joint age-group-by-condition interaction did not reach the conventional significance level, robust score χ^2^ (9) = 16.59, *p* = 0.056. In the final additive model, the overall age-group effect was significant, bias-reduced Wald χ^2^ (9) = 37.01, *p* < 0.001, whereas the overall irony-condition effect was not, bias-reduced Wald χ^2^ (3) = 0.58, *p* = 0.901.

Bonferroni-adjusted joint comparisons across thresholds showed that the 10-year-old group differed significantly from the 6-year-old group, adjusted *p* < 0.001, and from the 8-year-old group, adjusted *p* = 0.043. None of the remaining comparisons reached the 0.05 significance level: 6 versus 7 years, adjusted *p* = 0.062; 6 versus 8 years, adjusted *p* = 0.153; 7 versus 8 years, adjusted *p* = 0.254; and 7 versus 10 years, adjusted *p* = 0.075. These tests concern differences in the overall cumulative response distributions and do not imply a uniform shift across all comprehension thresholds.

Threshold-specific odds ratios are presented in Table S1 of the Supplementary Materials. Relative to the 6-year-old group, the odds ratios for the 10-year-old group were 3.80 for attaining at least Level 1, 7.37 for attaining at least Level 2, and 30.19 for attaining Level 3. Relative to the 8-year-old group, the corresponding odds ratios for the 10-year-old group were 1.95, 3.42, and 3.54. The confidence intervals were wide for some comparisons, particularly those involving Level 3, and these estimates should therefore be interpreted cautiously.

**Figure 1:**
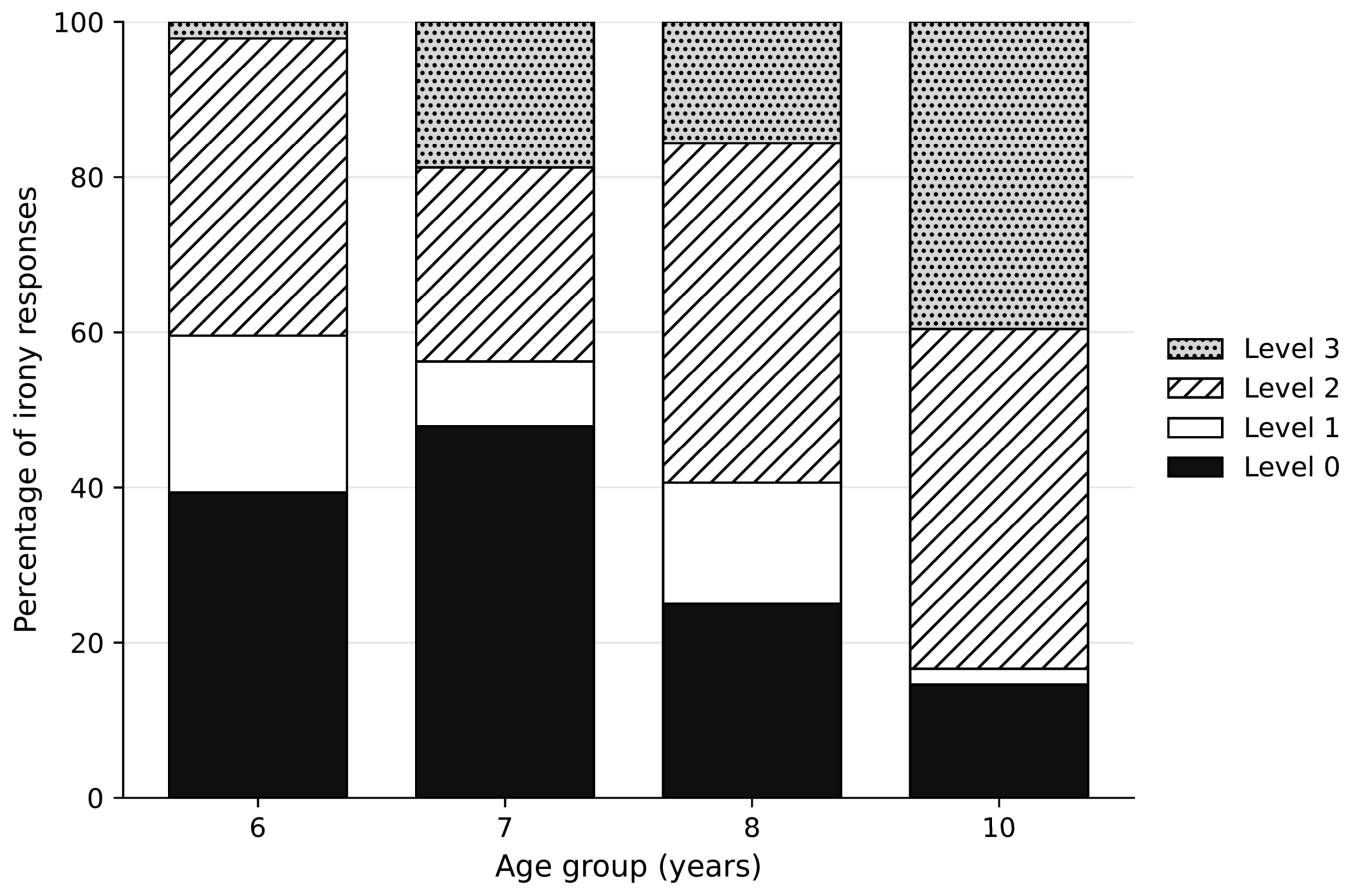
Percentage distribution of irony-comprehension levels by age group. Note. Percentages were calculated from 94, 96, 96, and 48 classified individual irony-story responses in the 6-, 7-, 8-, and 10-year-old groups, respectively.

Descriptively, the mean coded level increased from 1.03 at age 6 to 1.15 at age 7, 1.50 at age 8, and 2.08 at age 10. Figure 1 shows that responses at Levels 2 and 3 were already present in the younger groups, together accounting for 40.4% of the classified responses at age 6 and 43.8% at age 7. Their combined proportion increased to 59.4% at age 8 and 83.3% at age 10, indicating that higher coded response levels were more frequent among older children in these two stories. This shift was not uniform across individual levels, however. Level 2 was the most frequent category at both ages 8 and 10 (43.8% in each group), whereas Level 3 accounted for 18.8% of responses at age 7, 15.6% at age 8, and 39.6% at age 10. Moreover, responses at Levels 0 and 1 remained present even at age 10. The coexistence of different response levels within each age group, together with their changing distribution across ages, suggests substantial variability in children’s irony comprehension and possible differences in the development of meaning inference and explicit explanation.

To examine whether the treatment of near-correct explanations affected the results, the generalized ordinal GEE analysis was repeated after all 19 near-correct responses were reassigned from Level 3 to Level 2. The overall age-group effect remained significant, Wald χ^2^ (9) = 35.96, *p* < 0.001, whereas the irony-condition effect, Wald χ^2^ (3) = 0.33, *p* = 0.955, and the age-group-by-condition interaction, score χ^2^ (9) = 12.09, *p* = 0.209, remained nonsignificant. The adjusted comparisons between the 10- and 6-year-old groups (*p* < 0.001) and between the 10- and 8-year-old groups (*p* = 0.043) also remained significant. The comparison between the 8- and 7-year-old groups additionally reached significance (*p* = 0.045). Thus, the overall age-related pattern and the two principal pairwise differences identified in the primary analysis did not depend on the treatment of the near-correct explanations, although the 7–8-year comparison was sensitive to this coding decision.

### 3.4. Error Analyses in Children’s Comprehension of Irony

#### 3.4.1. Classification of Error Types

The answers to the explanation question associated with responses classified at Levels 0–2 were examined to characterize unsuccessful interpretations of irony. Five recurring response categories were derived inductively from the children’s answers during preliminary coding (Table 5). One author identified recurring patterns in the responses and proposed the initial category definitions, and a second author reviewed the preliminary coding and category definitions, with disagreements or uncertain cases resolved through discussion. This classification was not independently double-coded, and no inter-coder reliability statistic was calculated for these categories.

When explaining the utterance, children who gave Type 2 responses altered facts stated in the story, making the utterance’s literal meaning consistent with their account of events. Children who gave Type 1 responses introduced or assumed information not stated in the story about events, perceptions, or characters’ intentions concerning those events.

Type 3 responses relied on the literal wording without adding new information or altering the story facts. Type 4 responses attributed the utterance to the speaker’s attempt to conceal a negative attitude or manage the social consequences of direct criticism. Because this category includes politeness, conflict avoidance, and relationship maintenance, it is described as a concealment-based interpretation rather than deception in the strict sense.

#### 3.4.2. Error Types by Response Level

To describe how error patterns were distributed across response levels, we tabulated the five categories separately for responses classified at Levels 0, 1, and 2 (Table 6).

Fabrication of new information accounted for 37.7%, 30.2%, and 10.6% of responses at Levels 0, 1, and 2, respectively. The 13 Level 2 responses in this category combined correct judgments of dissatisfaction and criticism with explanations referring to verbal or perceptual errors, a favorable appraisal of the outcome, helpfulness, or lack of harmful intent. Examples included “She did not see the whole thing” and “He did not do it on purpose.” Some explanations were inconsistent with the criticism judgment; others offered a reason for the remark without explaining its ironic meaning. Concealment-based interpretations accounted for 6.1%, 25.6%, and 35.8% of responses at the three levels, respectively. At Level 2, no relevant explanation was the most frequent outcome (53.7%).

These distributions should also be interpreted in light of the classification procedure. Response levels and error types were not independent: level classification used the attitude-detection, meaning-inference, and explanation responses, whereas error types were derived from the explanation responses. Differences across levels are therefore partly constrained by the classification criteria, and Table 6 describes the composition of the response categories rather than providing independent evidence for a developmental sequence.

## 4. Discussion

### 4.1. Irony-Comprehension Response Levels Across Age Groups

The present study combines measures of attitude detection, meaning inference, and explicit explanation into an explicit hierarchical classification comprising four ordered performance levels. The aggregated response patterns across the three measures were consistent with this ordering. Of the 179 responses that were correct on meaning inference, 98.9% were also correct on attitude detection; among the 116 responses that were incorrect on attitude detection, only 1.7% were correct on meaning inference. Of the 45 responses showing inconsistent outcomes across these two tasks, 43 involved correct attitude detection but incorrect meaning inference, whereas only two showed the reverse pattern. Although the proposed ordering characterized nearly all observed responses, these two exceptions show that the four-level classification does not accommodate every combination of attitude and meaning judgments. Among the 179 responses that were correct on meaning inference, 30.2% were accompanied by correct or near-correct explanations, 31.9% by incorrect explanations, and 38.0% by no explanation. These levels describe graded differences in task performance rather than fixed developmental stages:

Level 0 (pre-understanding level). Responses classified at Level 0 contained incorrect judgments of both the speaker’s attitude and the utterance meaning. Explanations accompanying Level 0 responses included sincere or literal interpretations of the utterance. For example, a Level 0 response from a 6-year-old participant (Jin) was accompanied by the following explanation:

“Xiaohong originally wanted to knock down the block house but did not want to do it herself. Now that Xiaoming has knocked it down, Xiaohong is very satisfied and is praising him. After hearing what Xiaohong said, Xiaoming felt that he had done a good job and was very happy.”

Jin’s explanation preserved a literal interpretation by adding an intention that was not stated in the story. Some Level 0 explanations nevertheless showed sensitivity to the discrepancy between the literal utterance and the story outcome. For example, another 6-year-old participant (Li), whose response was also classified at Level 0, expressed the following confusion:

“Xiaoming knocked down the block house, so Xiaohong should be unhappy. Why did she still say, ‘You did a great job’?”

This example suggests that Li noticed the discrepancy but did not resolve it by inferring the intended ironic meaning. Other Level 0 explanations maintained a literal interpretation by adding information or altering the stated facts.

Level 1 (attitude detection level). Responses classified at Level 1 contained a correct judgment of the speaker’s attitude but an incorrect judgment of the utterance meaning. For example, a Level 1 response from a 7-year-old participant (Wang) was accompanied by the following explanation:

“Xiaohong was unhappy in her heart, but she did not dare to say so. They are good friends, and if she criticized Xiaoming, he would not play with her anymore, so she had to pretend to praise him.”

This response combined correct detection of the speaker’s displeased attitude with an incorrect judgment of the utterance meaning. Explanations accompanying Level 1 responses sometimes reconciled this conflict by adding information or construing the utterance as a means of concealing the speaker’s negative attitude.

Level 2 (Meaning inference level). Responses classified at Level 2 contained correct judgments of both the speaker’s attitude and the utterance meaning but did not meet the explanation criterion. For example, a Level 2 response from an 8-year-old participant (Zheng) included the following:

“Xiaohong was very angry and thought to herself, ‘Next time I won’t let him come and mess things up again.’ Xiaoming was criticized by Xiaohong and felt very sad.”

However, when asked, “Why did Xiaohong say that?” this participant replied, “Xiaohong was deceiving Xiaoming.” This example illustrates that the response correctly identified the speaker’s attitude and intended criticism but did not explain the relation between the literal praise and the intended meaning.

Level 3 (verbal explanation level). Responses classified at Level 3 contained correct judgments of the speaker’s attitude and utterance meaning together with a correct or near-correct explanation. For example, a Level 3 response from a 10-year-old participant (also named Wang) included the following explanation:

“Xiaohong was very angry. She was criticizing Xiaoming and speaking in the opposite way. My mother sometimes talks that way too. One time I was playing video games and my mother said, ‘Go ahead and play. That way you will be successful and never have to go to school again.’”

This explanation explicitly connected the intended criticism with an opposite form of expression and related it to a familiar example. Level 3 explanations included exact labels such as “saying the opposite” and “speaking in the opposite direction,” as well as related descriptions without an exact label, such as “using an opposite word” and “‘You did a great job’ can also be a criticism.” Near-correct explanations in this category referred to the speaker’s negative emotion or marked tone, without explicitly identifying the discrepancy between the utterance’s apparent and intended meanings.

The generalized ordinal GEE analysis showed a significant overall age-group effect on the cumulative distribution of response levels. After Bonferroni adjustment, the 10-year-old group differed significantly from the 6-year-old and 8-year-old groups, whereas the remaining pairwise comparisons were not significant. Because the proportional-odds assumption was not supported, the age-group differences did not represent a uniform shift across all three cumulative thresholds. Descriptively, responses classified at Levels 2 and 3 together accounted for 40.4% of the classified responses at age 6, 43.8% at age 7, 59.4% at age 8, and 83.3% at age 10. Level 2 was the most frequent response category at both ages 8 and 10, accounting for 43.8% of responses in each group. Level 3 accounted for 18.8% of responses at age 7, 15.6% at age 8, and 39.6% at age 10, and responses at Levels 0 and 1 remained present at age 10. The greater frequency of higher-level responses among older children may partly reflect the accumulation of linguistic experience and broader opportunities for peer communication across middle childhood.

The coexistence of multiple response levels within every age group indicates that irony comprehension was not an all-or-none outcome in the present task. Responses at Levels 2 and 3 together were more frequent in the older age groups, although the pattern varied across individual levels and age comparisons. The findings therefore support age-related differences in the frequency of response profiles; longitudinal evidence would be required to determine whether individual children pass through these levels sequentially. Related research likewise shows that children around age eight vary in whether they explicitly reference speakers’ intentions, emotions, and pragmatic conventions when explaining irony (Garfinkel et al., 2024). Forced-choice meaning-inference responses may not fully capture children’s sensitivity to insincerity in the attitudinal-irony condition; this measurement constraint should be considered when interpreting the response levels.

### 4.2. Error Types Across Response Levels

Previous research has rarely described the composition of irony-comprehension errors across response levels. In the present data, fabrication, distortion, and literal reliance together accounted for 62.3% of Level 0 responses, 41.9% of Level 1 responses, and 10.6% of Level 2 responses. Concealment-based interpretations accounted for 6.1%, 25.6%, and 35.8%, respectively. These patterns are descriptive and, because response levels and error types were derived from overlapping task indicators, should not be treated as independent evidence for a developmental sequence.

At Levels 0 and 1, the literal meaning remained central to some unsuccessful interpretations: explanations added information, altered story facts, or relied on the literal wording. Concealment-based responses showed a different pattern, attributing the utterance to another communicative purpose, such as avoiding direct criticism or managing the social relationship, without identifying ironic intent. At Level 2, correct judgments of dissatisfaction and criticism could coexist with explanations that were inconsistent with those judgments or did not explain how the utterance conveyed criticism.

No relevant explanation accounted for 31.6% of Level 0 responses, 32.6% of Level 1 responses, and 53.7% of Level 2 responses. Such responses may reflect difficulty explaining the utterance, reluctance to offer an explanation when uncertain, or the expressive and metapragmatic demands of the task.

Analyses of error types can help characterize children’s difficulties in irony comprehension. One influential view holds that preschool children’s difficulty in understanding irony primarily lies in their inability to carry out the second-order mental-state inference required for comprehension of irony, such as “Xiaohong does not want Xiaoming to believe what she says” (Winner & Leekam, 1991). According to this view, the key distinction between deception and irony lies in the difference in second-order intentions (whether Xiaohong wants Xiaoming to believe her utterance). Therefore, the main error type in children’s comprehension of irony should be interpreting irony as deception.

Across the 280 responses classified at Levels 0–2, 62 (22.1%) were coded as concealment-based interpretations, and 44 of these 62 responses occurred at Level 2. Concealment-based interpretations therefore constituted only one of several forms of unsuccessful interpretation, alongside fabrication, distortion, literal reliance, and absent explanations. These categories characterize the forms of unsuccessful interpretation rather than the cognitive processes that produced them.

Research with adults further shows that higher scores on the standard theory-of-mind “Reading the Mind in the Eyes” test are associated with a greater tendency to give ironic rather than literal interpretations of utterances, whereas this theory-of-mind advantage does not affect comprehension of literal sentences (Ronderos et al., 2024). Studies of children with autism likewise show heterogeneity in the relation between theory-of-mind task performance and irony comprehension. Children performing at ceiling on the irony task scored higher on average on first- and second-order theory-of-mind tasks than children who rarely understood irony, yet some children showed high irony-task performance despite failing both theory-of-mind tasks (Panzeri et al., 2022). These findings highlight possible dissociations between performance on standard theory-of-mind tasks and irony-comprehension tasks at the individual level.

### 4.3. Limitations and Suggestions for Future Research

The present findings indicate that Mandarin-speaking children’s irony comprehension shows age-related differences, but these differences do not constitute a uniform year-by-year pattern. Descriptively, higher-level responses were more frequent among older children, whereas multiple response levels continued to coexist within each age group. This pattern is broadly consistent with age-related developmental differences reported in previous research (Ackerman, 1983).

Several limitations of the present design constrain the interpretation and generalizability of the findings. First, the study used a cross-sectional design and examined only four age groups, with unequal sample sizes across groups and a relatively small 10-year-old group. Therefore, the present age-group differences cannot directly demonstrate within-child developmental change over time. Second, with respect to the materials, the study used only two irony stories based on the same block-building scenario, and thus covered a limited range of ironic forms and communicative contexts. Although all children received the same recordings and pictures within each story, prosody was not established as equivalent across stories. In addition, the four stories used similar characters and event structures, and the literal and deception stories were always presented before the irony stories. Although the order of the two irony stories was counterbalanced, these procedural arrangements may still have influenced children’s expectations about subsequent stories. Finally, with respect to measurement, the forced-choice meaning-inference task may not have fully distinguished children’s recognition of insincerity, inference of speaker intention, and judgment of the utterance’s evaluative meaning. The classification of Level 3 relied on children’s verbal explanations, yet the study did not include independent measures of language ability or metapragmatic ability. Some procedural information, such as the number of story replays and the prompts used to elicit children’s explanations, was also not fully documented. These limitations mean that whether the response profiles observed in the present study can be generalized to other materials, task measures, and testing procedures remains to be further examined.

These limitations point to several direct directions for future research. First, future studies could adopt longitudinal designs, increase and balance the sample sizes across age groups, and extend the age range beyond 10 years in order to further clarify within-child developmental trajectories in irony comprehension. Second, future research should use a more diverse set of irony materials, systematically controlling or manipulating prosodic cues while varying communicative context, utterance form, and degree of obviousness. This would help determine whether the within-age individual differences observed in the present study are stable across different types and difficulty levels of irony tasks, and would also clarify how irony difficulty affects children’s comprehension performance (Hancock et al., 2000). Cross-linguistic and cross-cultural studies using matched materials and procedures could further examine whether the age-related response distributions found in the present study generalize beyond Mandarin-speaking children. Future studies should also include independent measures of language ability and metapragmatic ability, so as to distinguish children’s performance on explicit verbal explanation tasks more clearly from irony comprehension itself.

More broadly, children’s difficulties in comprehending irony are likely to be influenced by multiple factors, including knowledge and communicative experience, mental-state reasoning, executive functions, paralinguistic and contextual cues, and cultural background (Aguert, 2022; Brugman & Burgers, 2021; Cui, 2022; Hauptman et al., 2023; Pfeifer & Lai, 2021). Future research should measure these factors independently and further examine their independent and interactive contributions to individual differences in children’s irony comprehension. On this basis, intervention studies may also serve as a complementary approach to testing the role of metapragmatic knowledge. Previous research has shown that brief metapragmatic training can improve some aspects of children’s sarcasm or irony comprehension (Lee et al., 2021; Olkoniemi et al., 2026). Future studies could separately examine the effects of training on attitude detection, meaning inference, and verbal explanation, and test whether these effects generalize to Mandarin-speaking children and to a broader range of ironic expressions, as well as whether they are maintained over a longer period of time.

## Figures and Tables

**Table 1:** Demographic characteristics of the participants.

Age Group	Boys (*n*)	Girls (*n*)	Mean Age (Years)	Min	Max	SD
6-year-olds	24	24	5.84	5.6	6.3	0.18
7-year-olds	24	24	6.95	6.6	7.3	0.22
8-year-olds	24	24	7.94	7.7	8.3	0.15
10-year-olds	12	12	9.95	9.6	10.3	0.26

**Table 2:** Percentages of individual irony-story responses meeting each task criterion by age group.

Age Group	Attitude Detection Task (%)	Meaning Inference Task (%)	Explanation Task (%)
6-year-olds	59	42	2
7-year-olds	52	44	19
8-year-olds	75	59	16
10-year-olds	85	83	40

Note: Percentages are based on individual irony-story responses (one per condition per child; *n* = 96 for each of the 6-, 7-, and 8-year-old groups and *n* = 48 for the 10-year-old group). Correct and near-correct explanations were both counted as meeting the explanation criterion.

**Table 3:** Classification of levels of children’s comprehension of irony.

Level (Label)	Criterion
Level 0: Pre-understanding level	Incorrect judgments of both the speaker’s attitude and the utterance meaning.
Level 1: Attitude detection level	Correct judgment of the speaker’s attitude, but incorrect judgment of the utterance meaning.
Level 2: Meaning inference level	Correct judgments of both the speaker’s attitude and the utterance meaning, but inability to provide an adequate explanation of irony.
Level 3: Verbal explanation level	Correct judgments of both the speaker’s attitude and the utterance meaning, accompanied by a correct or near-correct verbal explanation.

**Table 4:** Mean level of irony comprehension by age group.

Age Group	Number of Classified Responses	Minimum Level	Maximum Level	M	SD
6	94	0	3	1.03	0.93
7	96	0	3	1.15	1.21
8	96	0	3	1.50	1.04
10	48	0	3	2.08	1.01

**Table 5:** Operational definitions and examples of error types in irony comprehension.

Error Type	Operational Definition and Example
Type 1: Fabrication of new information	Introduces or assumes information not stated in the story about events, perceptions, or intentions when explaining the speaker’s remark. Example: “She originally wanted to knock it down and build it again.”
Type 2: Distortion of factual information	Changes or denies facts explicitly stated in the story so that the literal utterance can be treated as accurate. Example: “He made it more beautiful.”
Type 3: Literal reliance	Relies on or repeats the literal utterance without adding or altering story information and without resolving its conflict with the situation. Example: “She just said exactly that.”
Type 4: Concealment-based interpretation	Attributes the utterance to concealing a negative attitude, avoiding direct criticism or conflict, sparing the addressee’s feelings, or maintaining the relationship, without identifying ironic intent. Example: “She didn’t dare criticize him because she was afraid he would get angry and wouldn’t play with her anymore.”
Type 5: No relevant explanation	Provides no relevant explanatory information, including silence, “I don’t know,” or an irrelevant response. Example: “I don’t know.”

**Table 6:** Distribution of error types among responses classified at Levels 0–2, *n* (%).

Response Level	Fabrication of New Information	Distortion of Facts	Literal Reliance	Concealment-Based Interpretation	No Relevant Explanation
Level 0	43 (37.7)	18 (15.8)	10 (8.8)	7 (6.1)	36 (31.6)
Level 1	13 (30.2)	2 (4.7)	3 (7.0)	11 (25.6)	14 (32.6)
Level 2	13 (10.6)	0 (0.0)	0 (0.0)	44 (35.8)	66 (53.7)

Note. Percentages were calculated within each response level (Level 0, *n* = 114; Level 1, *n* = 43; Level 2, *n* = 123). Level 3 responses were not included because they met the explanation criterion; the two responses that could not be assigned a level were also excluded. The comparisons reported in the text are descriptive; no inferential tests were conducted.

## Data Availability

The data presented in this study are available on request from the corresponding author.

## Supplementary Materials

The following supporting information can be downloaded at: https://www.mdpi.com/article/10.3390/bs16101803/s1, Text S1: Story materials; Table S1: Threshold-specific odds ratios by age-group comparison.
