# Supplementary material for: The Development of Mandarin-Speaking Children’s Comprehension of Irony
Source: Behav Sci (Basel). 2026 Oct 2;16(10):1803. doi: 10.3390/bs16101803 (PMC13701572; doi:10.3390/bs16101803)
Supplement: Supplementary file 1 [file behavsci-16-01803-s001.zip › behavsci-4533725-supplementary.pdf]

Text S1. Story materials

Literal story

Xiaohong was playing with building blocks. She had built a very tall house out of blocks. Xiaoming came over and said to Xiaohong, "I will add one more block." After Xiaoming spoke, he added another block on top. Now Xiaohong's block house was even more beautiful than before. Xiaohong said to Xiaoming, "You did a great job."

Deception story

Xiaohong was playing with building blocks. She had built a very tall house out of blocks, and then she had to go out to do something. She asked Xiaoming to help her finish building the house. After Xiaohong left, Xiaoming kicked the block house into a complete mess. He said to Xiaopang, "I will give you a piece of candy. You must not tell Xiaohong." Xiaopang agreed. A little while later, Xiaopang went outside and met Xiaohong. Xiaohong asked Xiaopang, "Did Xiaoming finish building my block house?" Xiaopang said to Xiaohong, "He did a great job."

Assertive-type irony story

Xiaohong was playing with building blocks. She had built a very tall house out of blocks. Xiaoming came over and said to Xiaohong, "I will add one more block." After Xiaoming spoke, he added another block on top, and as a result Xiaohong's block house collapsed. Xiaohong said to Xiaoming, "You did a great job."

Attitudinal-type irony story

Xiaohong was playing with building blocks. She had built a very tall house out of blocks. Xiaoming came over and said to Xiaohong, "I will add one more block." After Xiaoming spoke, he added another block on top, and as a result Xiaohong's block house collapsed. Xiaohong said to Xiaoming, "Thank you very much."

Original Mandarin-Chinese versions

字面故事：

小红在玩积木，她搭起了很高的一座积木房子。小明走过来对小红说："我再加上一块积木。" 小明说完，就在上面又加了一块积木，现在小红的积木房子比原来更漂亮了。小红对小明说："你干得好极了。"

欺骗故事：

小红在玩积木，她搭起了很高的一座积木房子，然后就有事出去了，她让小明帮她继续搭完。小红走了以后，小明把积木房子踢得乱七八糟，他对小胖说："我给你一块糖，你千万不要告诉小红。" 小胖答应了。过了一会儿，小胖来到外面，遇见了小红。小红问小胖："小明把我的积木房子搭好了吗？" 小胖对小红说："他干得好极了。"

断言型反语：

小红在玩积木，她搭起了很高的一座积木房子。小明走过来对小红说："我再加上一块积木。" 小明说完，就在上面又加了一块积木，结果小红的积木房子倒掉了。小红对小明说："你干得好极了。"

表态型反语：

小红在玩积木，她搭起了很高的一座积木房子。小明走过来对小红说："我再加上一块积木。" 小明说完，就在上面又加了一块积木，结果小红的积木房子倒掉了。小红对小明说："非常感谢你。"

Table S1. Threshold-specific odds ratios by age-group comparison

Comparison	OR for ≥ Level 1 (95% CI)	OR for ≥ Level 2 (95% CI)	OR for Level 3 (95% CI)	Joint $\chi^2(3)$	Adjusted p
7 vs. 6	0.71 (0.31–1.58)	1.15 (0.52–2.55)	10.63 (2.17–52.02)	11.28	.062
8 vs. 6	1.95 (0.85–4.43)	2.15 (0.99–4.69)	8.53 (1.77–41.07)	9.31	.153
10 vs. 6	3.80 (1.07–13.54)	7.37 (2.26–23.99)	30.19 (6.05–150.75)	26.93	< .001
8 vs. 7	2.76 (1.22–6.25)	1.88 (0.87–4.05)	0.80 (0.30–2.15)	8.19	.254
10 vs. 7	5.39 (1.52–19.12)	6.43 (1.99–20.80)	2.84 (1.00–8.04)	10.87	.075
10 vs. 8	1.95 (0.54–7.00)	3.42 (1.07–10.92)	3.54 (1.28–9.76)	12.09	.043

Note. ORs > 1 indicate higher odds of attaining the indicated or higher level in the first-listed age group; CIs are unadjusted, and p values are Bonferroni-corrected.
